# Patient safety incidents in mental health residential services: a multicenter, crosssectional, survey-based study

**DOI:** 10.3389/fpsyt.2026.1810559

**Published:** 2026-04-13

**Authors:** Sophia Russotto, Andrea Conti, Luca Inguaggiato, Alice Masini, José Joaquin Mira, Massimiliano Panella

**Affiliations:** 1 Department of Translational Medicine, Università del Piemonte Orientale, Novara, Italy; 2 Doctoral Program in Sports and Health—Patient Safety Line, Universitas Miguel Hernandez, Alicante, Spain; 3 Anteo Impresa Sociale, Biella, Italy; 4 Atenea Research, FISABIO, Hermanos López de Osaba, Alicante, Spain; 5 Direzione Medica dei Presidi Ospedalieri, Azienda Ospedaliero-Universitaria di Alessandria, Alessandria, Italy

**Keywords:** long-term care, mental health, patient safety, patient safety incidents, psychiatry, territorial mental health safety

## Abstract

**Introduction:**

While patient safety in general hospitals is well-researched, residential mental health services have received less attention. Psychiatric patients and staff working in such facilities face unique and complex risks. This study aims to explore and describe the frequency and characteristics of patient safety incidents (PSIs) experienced by professionals working in a cohort of Italian mental health residential services.

**Methods:**

A multicenter, cross-sectional, questionnaire-based observational study was conducted involving workers from 68 psychiatric mental health services from seven different Italian provinces. A total of 159 respondents, including both healthcare and non-healthcare professionals, took part in the study. Participants reported incidents personally experienced (FHE) or reported by colleagues (CRE) over the previous 12 months, using a taxonomy adapted from the World Health Organization’s Conceptual Framework for the International Classification for Patient Safety. Data was analyzed using descriptive statistics and zero-inflated models to identify associations between respondent characteristics and reporting behaviors.

**Results:**

A total of 4,432 FHE and 4,807 CRE were reported. The behavior-related events were the most frequently reported, followed by incidents related to organizational or infrastructure issues, and medication errors. Secondary analyses suggested that facilities with more than ten employees had significantly higher rates of both FHE and CRE. Furthermore, residential facilities showed a higher incidence rate of CRE compared to non-residential ones, and non-healthcare workers reported witnessing fewer incidents to colleagues (28%) compared to healthcare workers. Significant variability was also observed across different provinces.

**Conclusion:**

Residential mental health services experience a high incidence of PSIs, suggesting a risk profile distinct from general hospital settings. The predominance of behavior-related incidents corroborates the hypothesis that primary risks are mainly driven by relational dynamics. The findings highlight that both healthcare and non-healthcare staff are exposed to significant risks, suggesting the need of inclusive and comprehensive safety interventions. Consequently, specific training programs focused on relational risk management, de-escalation, and empathic communication are essential for all workers employed in these settings.

## 1 Introduction

The foundational principle of all health care is to ‘first, do no harm.’ Yet, despite this guiding tenet, significant evidence reveals a widespread and preventable burden of patient harm across health systems (1, 2). This issue carries serious human, ethical, moral, and financial consequences. Unsafe medical care is a significant global public health issue, affecting millions of patients each year. Recent data suggested that over 10% of patients experience at least one adverse event during their hospitalization, with nearly half of these incidents causing more than moderate to severe harm (3). Alarmingly, up to 12% of such harm leads to permanent disability or even death. Recent data suggests that unsafe care is responsible for over 3 million deaths annually worldwide, and around half of these cases are considered preventable (3). Unsafe care in health care systems incurs significant financial and economic costs (3, 4). Patient safety is defined as the “avoidance, prevention and amelioration of adverse outcomes or injuries stemming from the process of healthcare” (5). The recent *Global Patient Safety Report* provides a foundational understanding of the current state of patient safety globally (3).

Although substantial research has focused on patient safety in general hospital settings, mental health inpatient facilities have received comparatively less attention—despite presenting distinct challenges and risks. including Patient Safety Incidents (PSIs), which are events or situations that could have, or did, lead to unnecessary harm to a patient (6). Even if interest in patient safety has grown in recent years, research specific to mental health care remains scarce. Psychiatric patients may be exposed to adverse events (such as self-harm, aggression, medication errors, and falls), partly due to their condition, which can affect communication, understanding, and the timely recognition of their clinical needs (7, 8). While numerous studies have explored various dimensions of health care quality, few have focused specifically on patient safety within mental health settings. In fact, fewer than 25% of countries worldwide have implemented patient safety initiatives in this area (3).

As known by the Authors, only few reviews have examined patient safety in a mental health context and described factors that influence patient safety (7–10). Moreover, the vast majority of research has been conducted in hospitals and clinical settings, leaving other contexts—such as long-term care, residential services, and home care underexplored, despite some studies suggests that PSI frequently occur also in these settings (8, 11–13). This cross-sectional study aims to investigate the frequency and characteristics of patient safety incidents among different categories of professionals within a cohort of residential mental health services in Italy. Materials and methods.

## 2 Methods

### 2.1 Study design, setting, and participants

We conducted a cross-sectional, questionnaire-based observational study. The study was designed and therefore reported adopting the Strengthening the Reporting of Observational Studies in Epidemiology (STROBE) guidelines (14). The study protocol was approved by the University of Eastern Piedmont Ethics Committee (Prot. No. 247/CE of 10/03/2025).

The questionnaire was proposed to healthcare and non-healthcare workers employed in psychiatric community care facilities managed by the Italian not-for-profit company Anteo Impresa Sociale. This company manages 338 services in 12 out of 20 Italian regions and employs more than 2270 workers for managing 5000 beds and a yearly base of 22.133 users. With specific regards to the psychiatric sector, it employs 381 workers, 68 services (mainly supported housing, day centers, and communities) in five different Italian regions, for a total of 715 beds and 1723 users in 2024 (15).

The participation in the present study was proposed to all the employees from the abovementioned psychiatric services in October 2024. Specifically, the invitation was sent by e-mail, and information posters were put in all the facilities. Before answering, participants were asked to give their informed consent for research purposes. The initial invitation was sent on October 1st, and reminders were sent on October 15th and 31st, 2024.

### 2.2 Questionnaire

The questionnaire was administered online using the Sogolytics platform in October 2024. Participation was voluntary and anonymous; no personal information useful to identify respondents were collected. Responders did not receive any compensation for the participation. The study population included all personnel, regardless of their specific role or contract type (e.g., full- or part-time, hired or freelancer), employed in the psychiatric services of Anteo. An invitation to participate was distributed by facility managers using different channels (i.e., one-to-one contacts, team meetings, mails, WhatsApp messages, and notices posted on the bulletin board) on October 1, 2024, and the questionnaire remained accessible for completion until the end of the month. Moreover, reminders were made at the middle and before the end of October.

The questionnaire (available in **Supplementary Material 1** in original language) was structured into three distinct sections: demographic information, patient safety incidents, and second victim phenomenon.

The demographic information section gathered general information about the participants, including age, gender, and professional role, as well as characteristics of the facilities in which they are employed, such as type, province, and number of staff members.

In the second section, participants were asked to report the number of PSIs they were directly or indirectly involved in, and PSI in which they were not involved but acknowledged by colleagues over the previous 12 months. Since, as known by the Authors, there are not psychiatry-specific PSI taxonomies available, PSI list and classification were developed on the basis of the World Health Organization’s (WHO) “Conceptual Framework for the International Classification for Patient Safety”, refined and adapted basing on psychiatry-specific literature (16). The final list comprised 39 different PSI, classified in five categories.

### 2.3 Statistical analysis

Statistical analysis was conducted using R 4.5.2 (R Core Team, Vienna, Austria). To ensure the quality and accuracy of the findings, the collected data were screened for outliers using the modified Z-score with a cut-off value of 3. This statistical method was adopted to identify and manage anomalous values that could significantly deviate from the dataset’s normal pattern and potentially distort the interpretation of the results (17, 18). The data and the analyses presented in this study were performed after the removal of these outliers.

We used descriptive statistics, such as medians and means, for the population characteristics. PSI were described with both the raw number or reported events (for both first-hand events (FHE) and colleague-reported events (CRE)) and the incidence (number of events per person per year).

As a secondary analysis, we built zero-inflated models to explore the association between the respondents’ characteristics and the willingness to report PSIs. This approach was selected as the data under analysis is characterized, due to its nature, by a high prevalence of zero values. This “excess zero” phenomenon is theoretically expected, as the zero counts likely arise from two distinct processes: (1) a potential “structural” zero group, comprising respondents who are not in a position to experience or learn about incidents (e.g., due to their role or tasks), and (2) a count group, comprising at-risk respondents who simply did not have an incident during the observation period.

Zero-inflated models combine a logistic model to predict the probability of a respondent belonging to the “always-zero” structural group (subsequently, an OR < 1 indicates a higher probability of reporting at least one event) and a count model to predict the number of incidents for those in the “at-risk” group. This dual-component structure allows to explore how respondent characteristics are associated with both the likelihood of being in a non-reporting context and the frequency of incidents among those at risk.

For this models, demographic variables were categorized as following: age (low/equal to 47 and higher based on the median of age distribution), number of workers in the facility (up to ten and more than ten), frequency of work in contact with patients (daily contact or less frequent contact), healthcare (i.e., educators, nurses, psychologists, doctors, psychiatric rehabilitation technician) or non-healthcare worker (i.e., social workers, healthcare assistants, cleaners, occupational therapists), residential (i.e., communities and assisted livings) and non-residential facilities (i.e., day centers, psychiatric rehabilitation centers, educational services, and work rehabilitation services). Specifically, we performed two types of models: i) to measure differences in responses among provinces (stratified by residential/nonresidential facility), and ii) to explore the association between the abovementioned demographic factors with responses (stratified by province).

## 3 Results

### 3.1 Demographic information

A total of 159 subjects, representing 43% of the total population (i.e., 370 workers) responded to the questionnaire. Detailed demographic information is shown in **Table 1**. More than two thirds of the respondents were female, with 73% of them in the 31–60 age group. The three most represented provinces were Torino, Biella, and Foggia. The most common professional profiles among participants were healthcare assistant (i.e., “operatore socio-sanitario”) and educator, representing 73% of the respondents. Moreover, the vast majority of participants (119, 74.8%) worked either in a psychiatric community or in a psychiatric assisted living facility. Finally, most respondents (100, 62.8%) reported daily contact with patients. For each category, outliers ranged from 0.0% to 13.2% and from 0.0% to 16.4% for FHE and CRE, respectively.

**Table 1:** Demographic information of the respondents.

Variable	N (%)
Gender	
Female	111(69.8)
Male	45 (28.3)
Other/I do not want to answer	3 (1.9)
Age (mean, %)	44.2 (12.2)
Profession	
Social worker	6 (3.8)
Cleaner	1 (0.6)
Service coordinator	2 (1.3)
Educator	55 (34.6)
Physiotherapist	1 (0.6)
Nurse	12 (7.6)
Physician	2 (1.3)
Healthcare assistant	61 (38.4)
Psychologist	8 (5.0)
Occupational therapist	1 (0.6)
Psychiatric rehabilitation technician	10 (6.3)
Facility type	
Psychiatric community	70 (44.0)
Psychiatric day center	26 (16.4)
Psychiatric assisted living	49 (30.8)
Psychiatric rehabilitation center	2 (1.3)
Psychiatric work rehabilitation	2 (1.3)
Psychiatric home services	6 (3.8)
Psychiatric educational services	4 (2.5)
Province	
Alessandria	25 (15.7)
Asti	3 (1.9)
Biella	43 (27.0)
Chieti	5 (3.2)
Foggia	31 (19.5)
Torino	46 (28.9)
Vercelli	6 (3.8)
Frequency of work in contact with patients	
Every day	100 (63)
One or more times per week	54 (34)
One or more times per month	4 (3)

### 3.2 First-hand events

The largest number of the 4,432 reported FHE (**Table 2**) belonged to the “Patient Behavior” category, followed by “Organizational or infrastructure-related incidents” and “Medication”.

**Table 2:** Results of the “patient safety incidents” section of the questionnaire.

Category	PSI	FHE			CRE		
		Events (N)	Incidence (events/person/year)	Outliers (%)	Events (N)	Incidence (events/person/year)	Outliers (%)
Patient Behavior	Noncompliant/Uncooperative/Obstructive	762	5.29	9.4	720	5.33	15.1
	Inconsiderate/Rude/Hostile/Inappropriate	636	4.61	13.2	614	4.62	16.4
	Risky/Reckless/Dangerous	308	2.03	4.4	468	3.12	5.7
	Problem with Substance Use/Abuse	97	0.66	6.9	177	1.20	7.5
	Harassment	61	0.39	1.3	76	0.48	1.3
	Discrimination/Prejudice	200	1.30	3.1	292	1.90	3.1
	Wandering/Absconding	159	1.09	8.2	153	1.08	10.7
	Intended Self Harm/Suicide	113	0.75	5.0	121	0.85	10.7
	Verbal Aggression	547	3.80	9.4	476	3.38	11.3
	Physical Assault	101	0.66	3.8	94	0.66	10.1
	Sexual Assault	3	0.02	0.0	10	0.06	3.1
	Aggression toward an Inanimate Object	221	1.50	7.5	282	1.97	10.1
	Death Threat	69	0.45	1.9	44	0.29	4.4
	Total	3.277	20.61	5.7	3527	22.18	8.4
Medication	Wrong Patient	6	0.04	1.9	8	0.05	1.9
	Wrong Drug	14	0.09	5.7	11	0.07	6.3
	Wrong Dose/Strength of Frequency	47	0.31	3.8	61	0.40	4.4
	Contraindication	2	0.01	0.6	2	0.01	0.0
	Wrong Storage	16	0.10	0.6	8	0.05	1.9
	Omitted Medicine or Dose	202	1.42	10.7	254	1.76	9.4
	Expired Medicine	2	0.01	0.0	11	0.07	0.0
	Adverse Drug Reaction	7	0.04	1.3	4	0.03	0.6
	Total	296	1.86	3.1	359	2.26	3.1
Patient accidents	Falls	117	0.79	6.3	144	0.98	7.5
	Threat to Breathing	22	0.15	9.4	31	0.22	11.3
	Other Specified Mechanism of Injury	43	0.28	2.5	58	0.38	3.8
	Total	182	1.14	6.1%	233	1.47	7.5
Organizational or infrastructure- related incidents	Matching of Workload Management	130	0.85	3.8%	126	0.83	5.0
	Bed/Service Availability/Adequacy	71	0.45	0.0%	24	0.15	0.0
	Human Resource/Staff Availability/Adequacy	71	0.46	1.9%	84	0.54	2.5
	Organization of Teams/People	86	0.57	5.0%	147	0.94	1.3
	Protocols/Policy/Procedure/Guideline	39	0.25	3.8%	44	0.29	3.8
	Infrastructure Non-Existent/Inadequate	42	0.29	7.5%	64	0.43	6.3
	Infrastructure Damaged/Faulty/Worn	113	0.78	8.8%	114	0.77	6.3
	Total	552	3.47	4.4%	603	3.79	3.6
Other incidents	Management	57	0.36	0.0%	28	0.18	1.3
	Documentation	8	0.05	3.1%	13	0.08	1.3
	Equipment	2	0.01	0.6%	2	0.01	0.0
	Nutrition	32	0.20	1.3%	12	0.08	1.9
	Healthcare acquired infections	1	0.01	0.0%	4	0.03	0.6
	Incidents related to oxygen and medical gases	0	0.00	0.0%	0	0.00	0.0
	Incident related to blood transfusion	0	0.00	0.0%	1	0.01	0.0
	Incident caused by staff behavior	25	0.16	0.6%	25	0.16	0.0
	Total	125	0.79	0.7%	85	0.53	0.6

FHE, first-hand events; CRE, colleague-reported events. Outliers indicate the proportion of the overall responses to the specific questions which were removed based on the Z-score.

The five most frequent FHE were, in order, “Non-compliant”, “Inconsiderate”, “Verbal”, “Risky” and “Inanimate Object”. The most frequent FHE for each category were: “Non-compliant” (Patient Behavior), “Omitted medicine” (Medication), “Workload” (Organizational or infrastructure-related incidents), “Falls” (Patient Accident) and “Management” (Other Incidents).

### 3.3 Colleague-reported events

**Figure 1:**
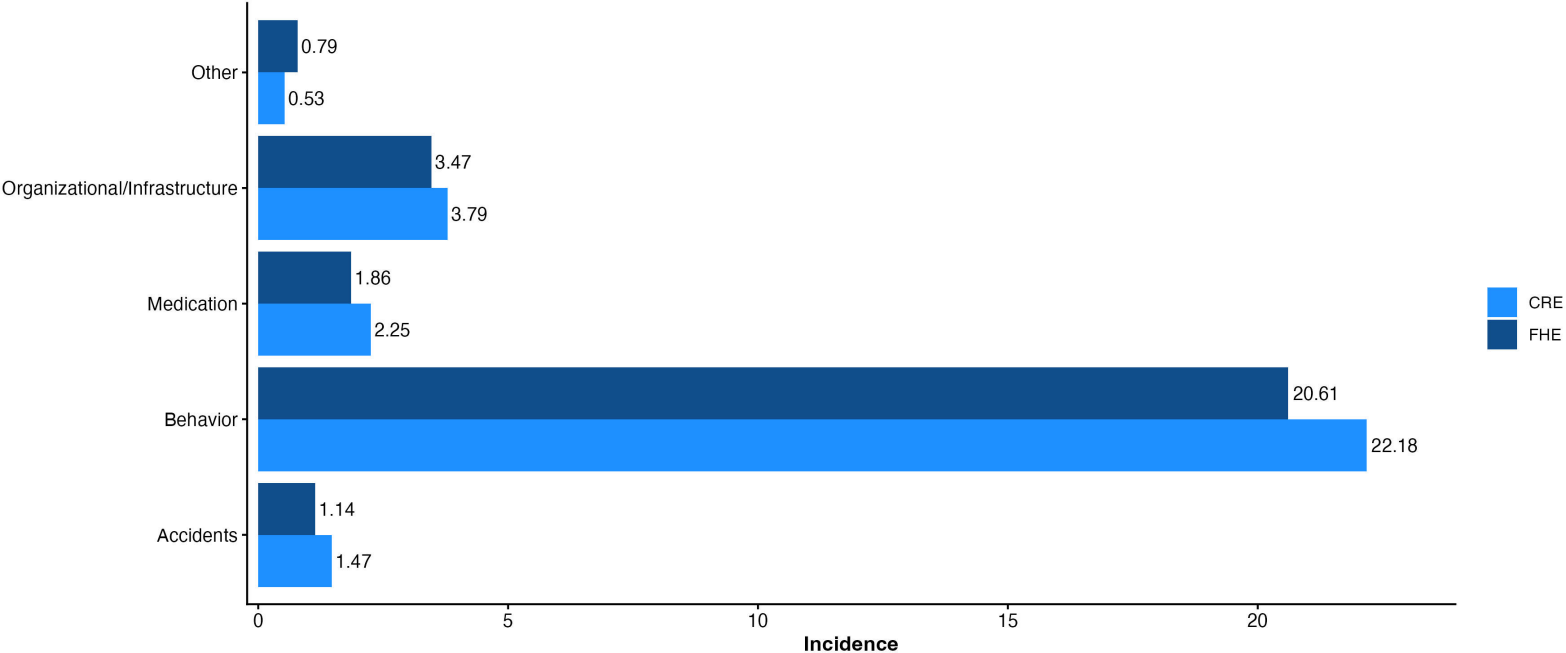
Incidence (events per person/year) of the different PSI categories.

A total of 4,807 CRE were recorded (**Table 2**). The highest incidence category was the “Patient Behavior” category, followed by “Organizational or infrastructure-related incidents” and “Medication”. The five most frequent CRE were, in order, “Non-compliant”, “Inconsiderate”, “Verbal”, “Risky” and “Inanimate Object” (**Table 2**). Lastly, the most represented CRE for each category were “Non-compliant” (Patient Behavior), “Omitted medicine” (Medication), “Falls” (Patient Accidents), “Organization” (Organizational or infrastructure-related incidents) and “Management” (Other Incidents) (**Figure 1**).

### 3.4 Secondary analysis

The secondary analyses aimed to identify association between respondent characteristics and the probability and frequency of being exposed to FHE and CRE. Results of the analyses are available in **Table 3** and in **Supplementary Material 2**. Facilities with ≤ 10 employees showed a significantly lower rate of FHE (IRR = 0.54, 95% CI: 0.40-0.72, p<0.001) compared to those with more than 10 employees. Moreover, residentials structures, compared to non-residentials facilities, showed a higher incidence rate of CRE (IRR = 1.52, 95% CI: 1.02-2.26, p=0.042). In addition, residentials facilities workers were more prone to reporting at least one FHE (OR = 0.12, 95% CI: 0.03-0.39, p=0.001) or CRE (OR = 0.15, 95% CI: 0.04-0.52, p=0.003) compared to workers from non-residential facilities. Also, CRE incidence was 28% lower in non-healthcare workers (IRR = 0.72, 95% CI: 0.54-0.96, p=0.025) when compared to healthcare workers. Lastly, every province (Asti, Chieti, Torino, Biella, Foggia) except Vercelli showed a statistically significant higher rate of incidence of both types of events compared to the province of Alessandria (details are available in **Table 3**). Furthermore, respondents from Biella in comparison to respondents in Alessandria showed a higher probability of reporting at least one PSI, both in the FHE (OR = 0.16, 95% CI: 0.03-0.72, p=0.018) and in the CRE (OR = 0.10, CI: 0.02-0.57, p=0.009) category. Similarly, respondents from Torino were more likely to report at least one CRE(OR = 0.08, CI: 0.01-0.82, p=0.034).

**Table 3:** Overall results of the zero-inflated models.

Facility location		First-hand events						Colleague-reported events					
		Count			Zero-inflated			Count			Zero-inflated		
		IRR	CI	p-value	IRR	CI	p-value	IRR	CI	p-value	IRR	CI	p-value
Facility location (province)	Alessandria (reference)	8.82 ^***^	5.75 – 13.53	<0.001	0.45	0.09 – 2.31	0.337	11.55 ^***^	7.26 – 18.35	<0.001	0.46	0.11 – 1.89	0.282
	Asti	6.89 ^***^	2.45 – 19.38	<0.001	0.00	0.00 – Inf	0.997	5.33 ^**^	1.84 – 15.49	0.002	0.00	0.00 – Inf	0.999
	Biella	3.82 ^***^	2.32 – 6.30	<0.001	0.16 ^*^	0.03 – 0.72	0.018	3.26 ^***^	1.96 – 5.41	<0.001	0.10 ^**^	0.02 – 0.57	0.009
	Chieti	5.89 ^***^	2.52 – 13.73	<0.001	0.00	0.00 – Inf	0.999	4.38 ^**^	1.74 – 11.00	0.002	0.00	0.00 – Inf	0.997
	Foggia	3.37 ^***^	1.99 – 5.73	<0.001	0.25	0.05 – 1.17	0.078	2.64 ^***^	1.52 – 4.61	0.001	0.35	0.09 – 1.44	0.146
	Torino	3.97 ^***^	2.42 – 6.51	<0.001	0.21	0.03 – 1.34	0.100	2.66 ^***^	1.53 – 4.61	<0.001	0.08 ^*^	0.01 – 0.82	0.034
	Vercelli	1.36	0.60 – 3.10	0.460	0.00	0.00 – Inf	0.998	0.94	0.40 – 2.22	0.882	0.00	0.00 – Inf	0.999
Profession	Healthcare worker (reference)	28.89 ^***^	17.77 – 46.97	<0.001	0.07 ^***^	0.02 – 0.21	<0.001	33.96 ^***^	21.85 – 52.78	<0.001	0.04 ^***^	0.01 – 0.19	<0.001
	Non-healthcare worker	0.89	0.66 – 1.19	0.430	1.82	0.62 – 5.37	0.279	0.72 ^*^	0.54 – 0.96	0.025	2.31	0.73 – 7.31	0.154
Facility type	Non residential (reference)	25.17 ^***^	14.44 – 43.89	<0.001	0.32 ^*^	0.12 – 0.87	0.026	21.77 ^***^	13.04 – 36.35	<0.001	0.23 ^*^	0.07 – 0.80	0.021
	Residential	1.13	0.75 – 1.69	0.564	0.12 ^***^	0.03 – 0.39	0.001	1.52 ^*^	1.02 – 2.26	0.042	0.15 ^**^	0.04 – 0.52	0.003
Age	48+ y.o. (reference)	28.55 ^***^	17.31 – 47.08	<0.001	0.07 ^***^	0.02 – 0.24	<0.001	27.60 ^***^	17.96 – 42.42	<0.001	0.04 ^***^	0.01 – 0.20	<0.001
	0–47 y.o.	0.94	0.70 – 1.26	0.662	1.47	0.49 – 4.46	0.494	1.14	0.84 – 1.53	0.397	2.01	0.61 – 6.63	0.254
Gender	Female (reference)	26.86 ^***^	16.58 – 43.51	<0.001	0.10 ^***^	0.03 – 0.27	<0.001	27.52 ^***^	17.61 – 43.00	<0.001	0.06 ^***^	0.02 – 0.22	<0.001
	Male	1.09	0.79 – 1.51	0.598	0.71	0.21 – 2.43	0.587	1.32	0.95 – 1.83	0.095	1.67	0.52 – 5.34	0.387
Frequency of patient contact	Daily (reference)	25.67 ^***^	15.79 – 41.71	<0.001	0.09 ^***^	0.03 – 0.27	<0.001	28.21 ^***^	18.41 – 43.23	<0.001	0.08 ^***^	0.02 – 0.29	<0.001
	Weekly or monthly	1.20	0.88 – 1.64	0.238	0.84	0.26 – 2.66	0.766	1.15	0.84 – 1.56	0.383	0.66	0.19 – 2.31	0.516
Number of workers employed in the facility	11+ (reference)	39.26 ^***^	23.67 – 65.12	<0.001	0.07 ^***^	0.02 – 0.24	<0.001	38.94 ^***^	25.09 – 60.45	<0.001	0.06 ^***^	0.01 – 0.25	<0.001
	0-10	0.54 ^***^	0.40 – 0.72	<0.001	1.50	0.49 – 4.62	0.479	0.62 ^**^	0.46 – 0.84	0.002	1.33	0.42 – 4.25	0.629

* p<0.05 ** p<0.01 *** p<0.001.

No statistically significant results were observed regarding age, gender, and frequency of contact with patients.

## 4 Discussion

The present cross-sectional study aimed to estimate the frequency and explore the characteristics of PSI in mental health residential services. To the best of the authors’ knowledge, this is among the first observational studies conducted in this specific setting (19). Moreover, in contrast with the majority of the published literature (7), this study adopts an *a priori* taxonomy (i.e., the ICPS) (20, 21) rather than deriving categories from identified events (8). In line with current literature, the majority of the events fall in the “Patient Behavior” category, followed by organizational and medication-related events (8, 10, 22, 23). The more frequent types (non-compliance, inconsiderate behavior, verbal aggression) could suggest that the potentially primary risk in these settings could not be linked to procedural or technological errors, as occurs in acute hospitals, but to relational dynamics between professionals and patients. This frequency suggests that the patient’s relational and behavioral dimension could represent one of the areas of greatest vulnerability in psychiatry, with respect to the most common technical or procedural errors in the clinic field (24, 25). Compared to general hospital evidence, PSI categories in psychiatry show a different risk profile, more focused on interactions and contextual dynamics than on medical procedures. This trend seems to align with the findings from our recent systematic review (8), which emphasize the unique nature of risks in psychiatry. The high incidence of behavioral events suggests the need for specific training programs for relational and behavioral risk management, including de-escalation, empathic communication, and aggression prevention (8). Training programs in de-escalation and aggression management have been shown to improve staff confidence, communication skills, and preparedness in handling patient behaviors, even if their direct impact on incident reduction remains variable across contexts (26).

Secondary analysis revealed significant differences related to facility characteristics: larger facilities (>10 employed staff) and residential services were found to be at greater risk. It is plausible that factors such as greater care complexity and the management of patients with more intensive needs in residential facilities contribute to this difference (27). Nevertheless, further studies are required, combining acuity and complexity measures, as well as organizational variables such as nurse staffing or missed care, to gain a better understanding of their role in patient outcomes and in the frequency of adverse events. Exploring these “non-medical factors”, which include psychosocial components such as self-management and self-care abilities, may help in the development of complex interventions for this setting (28).

When it comes to professional profiles, our analysis showed that both healthcare and non-healthcare workers are equally exposed to PSI. However, non-healthcare workers report them less frequently to colleagues. While we are not able to identify the determinants of these barriers, previous studies identified difficulties in communication on patient aspects in non-healthcare workers, which could be due to the already acknowledged style of communication (i.e., more informal and verbal where compared to healthcare professionals) (29, 30). Several literatures have explored the effects of poor communication on patient safety, showing that it can lead to an increased risk for adverse events, delays in treatment and medication errors (31) Interprofessional communication can be challenging due to differences in training, education and roles between healthcare professions and non-healthcare professionists (32). A potential solution to improve the skills and the knowledge of all the professional figures involved in the patient care can be the interprofessional collaboration. This strategy is an important component in providing health services to improve patient safety (33). These types of interventions should be embedded from the beginning of undergraduate health professions education to better prepare health care and non-healthcare professionals to communicate effectively with both patients and colleagues.

Surprisingly, no significant differences were found based on gender, age, or frequency of direct contact with patients, suggesting that the risk of PSIs in psychiatry appears to be distributed across personal categories, rather than being driven by individual characteristics.

The observed relevant variability across provinces suggest that territorial factors may play a relevant role in both the incidence and reporting of PSIs, which may be due to potential heterogeneity in patient profiles, workload, and specific regulations. In this sense, it is worth mentioning that all the facilities belong to a single not-for-profit company (i.e., Anteo Impresa Sociale), which has an established quality assurance system (i.e., ISO 9001) also including standardized procedures and protocols applied to all of its facilities. In contrast, the relevant heterogeneity of health systems, regulations, services, and mental health patients referral among different Italian regions and local health authorities (“Aziende Sanitarie Locali”), could have contributed to these differences (34–36). While it is not possible to draw a conclusive interpretation, we speculate that the higher probability of reporting at least one PSI among respondents from some provinces (e.g., Biella and Torino) may reflect either greater exposure to incidents or a stronger safety and reporting culture. In this sense, we advocate that higher reporting rates should not be interpreted solely as indicators of poorer safety performance but might also signal increased awareness and sensitivity to patient safety issues. Overall, these findings support the notion that patient safety is influenced not only by individual characteristics but also by system-level and contextual factors.

### 4.1 Limitations

Our study has several limitations. First, although nearly half of the targeted employees participated, the limited sample size may have affected the external generalizability of our findings. Second, our results relied on self-reported events from the past 12 months. While this approach has been used in previous studies (37, 38), we must acknowledge the potential for memory bias, whereby the most frequent events (i.e., behavior-related PSIs) may have become normalized within the psychiatric setting, leading to underestimation. Similarly, we cannot exclude respondent and social desirability biases, which have already been documented in patient safety research (39, 40). Furthermore, although the multi-center design enabled comparison across different provinces, all participating facilities belong to the same company. While protocols and policies are standardized across these facilities, variations in internal safety culture and local regulations may have influenced both the occurrence of PSIs and their reporting rates (41, 42).

## 5 Conclusion

Residential mental health services show a high incidence of PSI, with characteristics similar to clinical psychiatric facilities. Moreover, the predominance of behavior-related incidents—such as non-compliance and verbal aggression—may suggest that risks in this setting are more likely driven by relational dynamics than by procedural or care errors. N on-healthcare staff also face a significant risk of being involved in PSI, suggesting that safety interventions should be applied to all the workers, independently from their professional profile. Moreover, the variability among different territories highlights how the already well-known health care services heterogeneity in Italy could also have an impact on patient safety. Finally results from our study corroborate the hypothesis that patient safety is an issue extending far from the merely clinical context, embracing also other care settings characterized by a predominant component of social care. Future research should explore the development of patient safety improvement initiatives tailored not only to these territorial contexts, but also simultaneously targeting different professional profiles. Moreover, policy makers should consider the development of nationwide patient safety guidelines also including all social and long-term care services, in order to reduce variability among regions and provinces in terms of care delivery and patient safety.

## Data Availability

The raw data supporting the conclusions of this article will be made available by the authors, without undue reservation.

## Supplementary material

Supplementary Material 1 Questionnaire (in original Italian language).

Supplementary Material 2 Zero-inflated models.
