# Supplementary material for: Patient safety incidents in mental health residential services: a multicenter, crosssectional, survey-based study
Source: Front Psychiatry. 2026 Apr 13;17:1810559. doi: 10.3389/fpsyt.2026.1810559 (PMC13111304; doi:10.3389/fpsyt.2026.1810559)
Supplement: Supplementary Material 1 — Questionnaire (in original Italian language). [file DataSheet1.pdf]

Facility location (province)

	First-hand experience		
Predictors	*Incidence Rate Ratios*	*CI*	*p*
**Count Model**			
(Intercept) [Alessandria]	8.82 ***	5.75 – 13.53	**<0.001**
Province [Asti]	6.89 ***	2.45 – 19.38	**<0.001**
Province [Biella]	3.82 ***	2.32 – 6.30	**<0.001**
Province [Chieti]	5.89 ***	2.52 – 13.73	**<0.001**
Province [Foggia]	3.37 ***	1.99 – 5.73	**<0.001**
Province [Torino]	3.97 ***	2.42 – 6.51	**<0.001**
Province [Vercelli]	1.36	0.60 – 3.10	0.460
(Intercept)	4.39	3.10 – 6.91	
**Zero-Inflated Model**			
(Intercept) [Alessandria]	0.45	0.09 – 2.31	0.337
Province [Asti]	0.00	0.00 – Inf	0.997
Province [Biella]	0.16 *	0.03 – 0.72	**0.018**
Province [Chieti]	0.00	0.00 – Inf	0.999
Province [Foggia]	0.25	0.05 – 1.17	0.078
Province [Torino]	0.21	0.03 – 1.34	0.100
Province [Vercelli]	0.00	0.00 – Inf	0.998
**Random Effects**			
σ^2	0.53		
τ_{00} Residenziale	0.00		
N Residenziale	2		
Observations	159		
Marginal R^2 / Conditional R^2	0.347 / NA		

* *p<0.05* ** *p<0.01* *** *p<0.001*

	Reported by colleagues		
Predictors	*Incidence Rate Ratios*	*CI*	*p*
**Count Model**			
(Intercept) [Alessandria]	11.55 ***	7.26 – 18.35	**<0.001**
Province [Asti]	5.33 **	1.84 – 15.49	**0.002**
Province [Biella]	3.26 ***	1.96 – 5.41	**<0.001**
Province [Chieti]	4.38 **	1.74 – 11.00	**0.002**
Province [Foggia]	2.64 ***	1.52 – 4.61	**0.001**
Province [Torino]	2.66 ***	1.53 – 4.61	**<0.001**
Province [Vercelli]	0.94	0.40 – 2.22	0.882
(Intercept)	4.38	3.09 – 6.89	
**Zero-Inflated Model**			
(Intercept) [Alessandria]	0.46	0.11 – 1.89	0.282
Province [Asti]	0.00	0.00 – Inf	0.999
Province [Biella]	0.10 **	0.02 – 0.57	**0.009**
Province [Chieti]	0.00	0.00 – Inf	0.997
Province [Foggia]	0.35	0.09 – 1.44	0.146
Province [Torino]	0.08 *	0.01 – 0.82	**0.034**
Province [Vercelli]	0.00	0.00 – Inf	0.999
**Random Effects**			
σ^2	0.53		
$\tau_{00\ Residenziale}$	0.02		
ICC	0.04		
$N_{Residenziale}$	2		
Observations	159		
Marginal R^2 / Conditional R^2	0.274 / 0.299		

* *p<0.05* ** *p<0.01* *** *p<0.001*

Profession

Predictors	First-hand experience<br>Incidence Rate Ratios	CI	p
**Count Model**			
(Intercept) [Healthcare]	28.89 ***	17.77 – 46.97	**<0.001**
Profession [NonHealthcare]	0.89	0.66 – 1.19	0.430
(Intercept)	4.06	2.90 – 6.33	
**Zero-Inflated Model**			
(Intercept) [Healthcare]	0.07 ***	0.02 – 0.21	**<0.001**
Profession [NonHealthcare]	1.82	0.62 – 5.37	0.279
**Random Effects**			
σ^2	0.56		
τ_{00} Province	0.33		
ICC	0.37		
N Province	7		
Observations	159		
Marginal R^2 / Conditional R^2	0.004 / 0.372		

* p<0.05 ** p<0.01 *** p<0.001

Predictors	Reported by colleagues<br>Incidence Rate Ratios	CI	p
**Count Model**			
(Intercept) [Healthcare]	33.96 ***	21.85 – 52.78	**<0.001**
Profession [NonHealthcare]	0.72 *	0.54 – 0.96	**0.025**
(Intercept)	4.14	2.96 – 6.45	
**Zero-Inflated Model**			
(Intercept) [Healthcare]	0.04 ***	0.01 – 0.19	**<0.001**
Profession [NonHealthcare]	2.31	0.73 – 7.31	0.154
**Random Effects**			
σ^2	0.55		
τ_{00} Province	0.25		
ICC	0.32		
N Province	7		
Observations	159		
Marginal R^2 / Conditional R^2	0.033 / 0.338		

* p<0.05 ** p<0.01 *** p<0.001

Facility type

	First-hand experience		
Predictors	*Incidence Rate Ratios*	*CI*	*p*
**Count Model**			
(Intercept) [NonResidential]	25.17 ***	14.44 – 43.89	**<0.001**
Type [Residential]	1.13	0.75 – 1.69	0.564
(Intercept)	4.06	2.90 – 6.34	
**Zero-Inflated Model**			
(Intercept) [NonResidential]	0.32 *	0.12 – 0.87	**0.026**
Type [Residential]	0.12 ***	0.03 – 0.39	**0.001**
**Random Effects**			
σ^2	0.56		
τ_{00} Province	0.32		
ICC	0.37		
N Province	7		
Observations	159		
Marginal R^2 / Conditional R^2	0.003 / 0.370		

** p<0.05 ** p<0.01 *** p<0.001*

	Reported by colleagues		
Predictors	*Incidence Rate Ratios*	*CI*	*p*
**Count Model**			
(Intercept) [NonResidential]	21.77 ***	13.04 – 36.35	**<0.001**
Type [Residential]	1.52 *	1.02 – 2.26	**0.042**
(Intercept)	4.12	2.94 – 6.43	
**Zero-Inflated Model**			
(Intercept) [NonResidential]	0.23 *	0.07 – 0.80	**0.021**
Type [Residential]	0.15 **	0.04 – 0.52	**0.003**
**Random Effects**			
σ^2	0.55		
τ_{00} Province	0.25		
ICC	0.31		
N Province	7		
Observations	159		
Marginal R^2 / Conditional R^2	0.039 / 0.340		

** p<0.05 ** p<0.01 *** p<0.001*

Age

	First-hand experience		
Predictors	*Incidence Rate Ratios*	*CI*	*p*
**Count Model**			
(Intercept) [48+ y.o.]	28.55 ***	17.31 – 47.08	**<0.001**
AgeGroupBinomial [0-47 y.o.]	0.94	0.70 – 1.26	0.662
(Intercept)	4.05	2.89 – 6.31	
**Zero-Inflated Model**			
(Intercept) [48+ y.o.]	0.07 ***	0.02 – 0.24	**<0.001**
AgeGroupBinomial [0-47 y.o.]	1.47	0.49 – 4.46	0.494
**Random Effects**			
σ^2	0.56		
τ_{00} Province	0.33		
ICC	0.37		
N Province	7		
Observations	159		
Marginal R^2 / Conditional R^2	0.001 / 0.371		

* *p<0.05* ** *p<0.01* *** *p<0.001*

	Reported by colleagues		
Predictors	*Incidence Rate Ratios*	*CI*	*p*
**Count Model**			
(Intercept) [48+ y.o.]	27.60 ***	17.96 – 42.42	**<0.001**
AgeGroupBinomial [0-47 y.o.]	1.14	0.84 – 1.53	0.397
(Intercept)	3.93	2.83 – 6.02	
**Zero-Inflated Model**			
(Intercept) [48+ y.o.]	0.04 ***	0.01 – 0.20	**<0.001**
AgeGroupBinomial [0-47 y.o.]	2.01	0.61 – 6.63	0.254
**Random Effects**			
σ^2	0.57		
τ_{00} Province	0.21		
ICC	0.27		
N Province	7		
Observations	159		
Marginal R^2 / Conditional R^2	0.005 / 0.278		

* *p<0.05* ** *p<0.01* *** *p<0.001*

Gender

	First-hand experience		
Predictors	*Incidence Rate Ratios*	*CI*	*p*
**Count Model**			
(Intercept) [Female]	26.86 ***	16.58 – 43.51	**<0.001**
Gender [Male]	1.09	0.79 – 1.51	0.598
(Intercept)	4.06	2.90 – 6.32	
**Zero-Inflated Model**			
(Intercept) [Female]	0.10 ***	0.03 – 0.27	**<0.001**
Gender [Male]	0.71	0.21 – 2.43	0.587
**Random Effects**			
σ^2	0.56		
τ_{00} Province	0.33		
ICC	0.38		
N Province	7		
Observations	159		
Marginal R^2 / Conditional R^2	0.002 / 0.376		

* *p<0.05* ** *p<0.01* *** *p<0.001*

	Reported by colleagues		
Predictors	*Incidence Rate Ratios*	*CI*	*p*
**Count Model**			
(Intercept) [Female]	27.52 ***	17.61 – 43.00	**<0.001**
Gender [Male]	1.32	0.95 – 1.83	0.095
(Intercept)	4.07	2.92 – 6.31	
**Zero-Inflated Model**			
(Intercept) [Female]	0.06 ***	0.02 – 0.22	**<0.001**
Gender [Male]	1.67	0.52 – 5.34	0.387
**Random Effects**			
σ^2	0.56		
τ_{00} Province	0.28		
ICC	0.33		
N Province	7		
Observations	159		
Marginal R^2 / Conditional R^2	0.019 / 0.346		

* *p<0.05* ** *p<0.01* *** *p<0.001*

Frequency of patient contact

	First-hand experience		
Predictors	*Incidence Rate Ratios*	*CI*	*p*
**Count Model**			
(Intercept) [Daily]	25.67 ***	15.79 – 41.71	**<0.001**
WorkFrequencyBinomial [Weekly or monthly]	1.20	0.88 – 1.64	0.238
(Intercept)	4.12	2.93 – 6.45	
**Zero-Inflated Model**			
(Intercept) [Daily]	0.09 ***	0.03 – 0.27	**<0.001**
WorkFrequencyBinomial [Weekly or monthly]	0.84	0.26 – 2.66	0.766
**Random Effects**			
σ^2	0.55		
τ_{00} Province	0.33		
ICC	0.38		
N Province	7		
Observations	159		
Marginal R^2 / Conditional R^2	0.009 / 0.381		

* p<0.05   ** p<0.01   *** p<0.001

	Reported by colleagues		
Predictors	*Incidence Rate Ratios*	*CI*	*p*
**Count Model**			
(Intercept) [Daily]	28.21 ***	18.41 – 43.23	**<0.001**
WorkFrequency [Weekly or monthly]	1.15	0.84 – 1.56	0.383
(Intercept)	3.95	2.85 – 6.08	
**Zero-Inflated Model**			
(Intercept) [Daily]	0.08 ***	0.02 – 0.29	**<0.001**
WorkFrequency [Weekly or monthly]	0.66	0.19 – 2.31	0.516
**Random Effects**			
σ^2	0.56		
τ_{00} Province	0.24		
ICC	0.29		
N Province	7		
Observations	159		
Marginal R^2 / Conditional R^2	0.006 / 0.299		

* p<0.05   ** p<0.01   *** p<0.001

Number of workers employed in the facility

	First-hand experience		
Predictors	*Incidence Rate Ratios*	*CI*	*p*
**Count Model**			
(Intercept) [11+]	39.26 ***	23.67 – 65.12	**<0.001**
NWorkers [0-10]	0.54 ***	0.40 – 0.72	**<0.001**
(Intercept)	4.97	3.38 – 8.24	
**Zero-Inflated Model**			
(Intercept) [11+]	0.07 ***	0.02 – 0.24	**<0.001**
NWorkers [0-10]	1.50	0.49 – 4.62	0.479
**Random Effects**			
σ^2	0.50		
τ_{00} Province	0.34		
ICC	0.40		
N Province	7		
Observations	159		
Marginal R^2 / Conditional R^2	0.101 / 0.461		

* p<0.05   ** p<0.01   *** p<0.001

	Reported by colleagues		
Predictors	*Incidence Rate Ratios*	*CI*	*p*
**Count Model**			
(Intercept) [11+]	38.94 ***	25.09 – 60.45	**<0.001**
NWorkers [0-10]	0.62 **	0.46 – 0.84	**0.002**
(Intercept)	4.37	3.08 – 6.93	
**Zero-Inflated Model**			
(Intercept) [11+]	0.06 ***	0.01 – 0.25	**<0.001**
NWorkers [0-10]	1.33	0.42 – 4.25	0.629
**Random Effects**			
σ^2	0.54		
τ_{00} Province	0.22		
ICC	0.29		
N Province	7		
Observations	159		
Marginal R^2 / Conditional R^2	0.069 / 0.343		

* p<0.05   ** p<0.01   *** p<0.001
