# Supplementary material for: Patient safety incidents in mental health residential services: a multicenter, crosssectional, survey-based study
Source: Front Psychiatry. 2026 Apr 13;17:1810559. doi: 10.3389/fpsyt.2026.1810559 (PMC13111304; doi:10.3389/fpsyt.2026.1810559)
Supplement: Supplementary Material 2 — Zero-inflated models. [file DataSheet2.pdf]

Questionnaire (in original Italian language)

Sezione 1 - Dati demografici

1. Con quale genere ti identifichi?

a. Donna
b. Uomo
c. Non binario
d. Non so/Non voglio rispondere

2. Quanti anni hai? [numero]

____

3. Qual è la tua professione? [una sola risposta]

a. Infermiere
b. OSS
c. Psicologo
d. Educatore
e. Fisioterapista
f. Medico
g. Addetto alle pulizie
h. Altro (specificare)
i. Con quale frequenza svolgi il tuo lavoro a contatto diretto con gli utenti? (Selezionare un'opzione)
j. Ogni giorno
k. Più volte alla settimana
l. Una volta alla settimana
m. Più volte al mese
n. Fino ad una volta al mese
o. Non lavoro mai a contatto diretto con gli utenti
p. Altro (specifica)

4. In quale tipologia di servizio svolgi prevalentemente la tua attività lavorativa? [una sola risposta]

a. Servizio ambulatoriale
b. Centro diurno
c. Comunità
d. Servizio domiciliare
e. Gruppo appartamento
f. Riabilitazione lavorativa
g. Servizio educativo
h. Altro (specifica) __________

5. In quale provincia svolgi prevalentemente la tua attività lavorativa? [una sola risposta]

a. (lista delle province italiane)

6. Quanti operatori lavorano nel servizio in cui svolgi prevalentemente la tua attività lavorativa? [una sola risposta]

a. Meno di 5
b. 5-10
c. 11-20
d. Più di 20

Sezione 2 - Incidenti

Ti proponiamo ora una lista di eventi fortuiti o involontari che hanno il potenziale di causare un danno ai pazienti (patient safety incidents). Facendo riferimento alla struttura presso la quale svolgi in prevalenza la tua attività lavorativa, dovrai indicare quante volte negli ultimi 12 mesi ciascuno di questi incidenti:

- Ti ha coinvolto/a o a cui hai assistito
- Ti è stato riferito dai colleghi

Nel caso tu non ricordassi l'accadimento di uno o più incidenti, indica "0" nella casella corrispondente.

1. Incidenti legati al comportamento dei pazienti

Numero di incidenti nei quali sei stato coinvolto/a o a cui hai assistito (ultimi 12 mesi)

- [ ] Comportamento disobbediente/non collaborante/ostacolante
- [ ] Comportamento sconsiderato/maleducato/ostile/inappropriato
- [ ] Comportamento spericolato/pericoloso
- [ ] Uso/abuso di sostanze
- [ ] Molestie
- [ ] Comportamento discriminatorio
- [ ] Fughe o vagabondaggio
- [ ] Autolesionismo volontario/suicidio
- [ ] Aggressione verbale
- [ ] Aggressione fisica
- [ ] Aggressione sessuale
- [ ] Aggressione nei confronti di un oggetto
- [ ] Minaccia di morte
- [ ] Numero di incidenti che ti sono stati riferiti (ultimi 12 mesi)

2. Incidenti legati alla terapia farmacologica

Numero di incidenti nei quali sei stato coinvolto/a o a cui hai assistito (ultimi 12 mesi)

- [ ] Assunzione da parte del paziente sbagliato
- [ ] Assunzione del farmaco sbagliato
- [ ] Errore di dosaggio/quantità/frequenza/via di assunzione
- [ ] Assunzione di un farmaco controindicato
- [ ] Conservazione errata del farmaco
- [ ] Mancata assunzione
- [ ] Assunzione di un farmaco scaduto
- [ ] Reazione avversa

Numero di incidenti che ti sono stati riferiti (ultimi 12 mesi)

- [ ] Assunzione da parte del paziente sbagliato
- [ ] Assunzione del farmaco sbagliato
- [ ] Errore di dosaggio/quantità/frequenza/via di assunzione
- [ ] Assunzione di un farmaco controindicato
- [ ] Conservazione errata del farmaco
- [ ] Mancata assunzione
- [ ] Assunzione di un farmaco scaduto
- [ ] Reazione avversa

3. Incidenti legati a eventi traumatici

Numero di incidenti nei quali sei stato coinvolto/a o a cui hai assistito (ultimi 12 mesi)

- ☐ Caduta del paziente
- ☐ Ostacolo alla respirazione (es. soffocamento)
- ☐ Altri eventi con trauma fisico del paziente

Numero di incidenti che ti sono stati riferiti (ultimi 12 mesi)

- ☐ Caduta del paziente
- ☐ Ostacolo alla respirazione (es. soffocamento)
- ☐ Altri eventi con trauma fisico del paziente

4. Incidenti legati a problematiche organizzative o strutturali

Numero di incidenti nei quali sei stato coinvolto/a o a cui hai assistito (ultimi 12 mesi)

- ☐ Carico di lavoro non adeguato
- ☐ Indisponibilità/inadeguatezza di posti letto o di servizi
- ☐ Indisponibilità/inadeguatezza del personale
- ☐ Inadeguatezza dell'organizzazione
- ☐ Indisponibilità/inadeguatezza di protocolli, procedure o linee guida
- ☐ Indisponibilità/inadeguatezza di infrastrutture, strutture o apparecchiature
- ☐ Malfunzionamento/usura di infrastrutture, strutture o apparecchiature

Numero di incidenti che ti sono stati riferiti (ultimi 12 mesi)

- ☐ Carico di lavoro non adeguato
- ☐ Indisponibilità/inadeguatezza di posti letto o di servizi
- ☐ Indisponibilità/inadeguatezza del personale
- ☐ Inadeguatezza dell'organizzazione
- ☐ Indisponibilità/inadeguatezza di protocolli, procedure o linee guida
- ☐ Indisponibilità/inadeguatezza di infrastrutture, strutture o apparecchiature
- ☐ Malfunzionamento/usura di infrastrutture, strutture o apparecchiature
- ☐

Altri incidenti

Numero di incidenti nei quali sei stato coinvolto/a o a cui hai assistito (ultimi 12 mesi)

- ☐ Incidenti legati alla gestione e coordinamento dei servizi e delle iniziative
- ☐ Incidenti legati alla documentazione
- ☐ Incidenti legati ai dispositivi ed equipaggiamenti medici
- ☐ Incidenti legati alla nutrizione/alimentazione degli ospiti
- ☐ Infezioni correlate all'assistenza
- ☐ Incidenti legati ai gas medicali
- ☐ Incidenti legati alle emotrasfusioni e agli emoderivati
- ☐ Incidenti legati al comportamento degli operatori
- ☐

Numero di incidenti che ti sono stati riferiti (ultimi 12 mesi)

- ☐ Incidenti legati alla gestione e coordinamento dei servizi e delle iniziative
- ☐ Incidenti legati alla documentazione
- ☐ Incidenti legati ai dispositivi ed equipaggiamenti medici
- ☐ Incidenti legati alla nutrizione/alimentazione degli ospiti
- ☐ Infezioni correlate all'assistenza
- ☐ Incidenti legati ai gas medicali
- ☐ Incidenti legati alle emotrasfusioni e agli emoderivati
- ☐ Incidenti legati al comportamento degli operatori
